# Hemiplegic-Migraine–like Attacks as First Manifestation of Diffuse Leptomeningeal Glioneuronal Tumor: A Case Report

**DOI:** 10.1097/MPH.0000000000002287

**Published:** 2021-09-03

**Authors:** Anna Fetta, Jacopo Pruccoli, Giacomo Biasucci, Roberto Parisi, Francesco Toni, Fraia Melchionda, Duccio M. Cordelli

**Affiliations:** * IRCCS Istitito delle Scienze Neurologiche di Bologna, UOC di Neuropsichiatria dell’età Pediatrica; ‡ Neuroradiology Department, IRCCS Institute of Neurological Sciences; § Pediatric Oncology and Hematology Unit ‘Lalla Seràgnoli’, Department of Medical and Surgical Sciences (DIMEC), S. Orsola Hospital, University of Bologna, Bologna; † Pediatrics and Neonatology Unit, Mother and Child Health Department, Guglielmo da Saliceto Hospital, AUSL, Piacenza, Italy

**Keywords:** hemiplegic migraine, symptomatic hemiplegic migraine, cortical spreading depression, DLGNT, leptomeninges, meningeal enhancement

## Abstract

Diffuse leptomeningeal glioneuronal tumor (DLGNT) is a low-grade tumor characterized by diffuse leptomeningeal infiltrates. Symptoms are usually secondary to hydrocephalus. Hemiplegic migraine (HM)-like episodes have never been associated with DLGNT, but they have been reported with different inflammatory and tumoral entities involving leptomeninges.

**Observations:**

We report the case of a 10-year-old boy with recurrent episodes of right hyposthenia, aphasia, and headache lasting hours to days with complete remission. The electroencephalogram during the attack showed diffuse slower activity on the left hemisphere, which improved together with the symptoms. DLGNT was discovered during a follow-up magnetic resonance imaging and confirmed by biopsy.

**Conclusions:**

This is the first report of HM-like attacks in DLGNT. We discuss the pathogenetic hypotheses of our case and previously reported cases of “symptomatic” HM with leptomeningeal involvement.

Hemiplegic migraine (HM) is a clinically and genetically heterogenous condition characterized by attacks of headache and motor weakness often associated with visual or sensory manifestations, aphasia, impaired consciousness, or brainstem aura. Symptoms usually last <72 hours. It can occur sporadically or in a familial form (FHM), associated or not with known genes mutation such as CACNA1A, ATP1A2, SCN1A. PRRT2 related FHM has been also reported.^1^,^2^

Diffuse leptomeningeal glioneuronal tumor (DLGNT) is a rare, recently classified, central nervous system neoplasm causing superficial infiltration of the leptomeninges and formation of subpial cysts without parenchymal involvement. A tendency to be indolent is reported, although a grade has not been assigned yet.

Typical onset symptoms are headache and vomiting secondary to communicating hydrocephalus.^3^ HM-like episodes have been occasionally associated with different conditions with leptomeningeal involvement,^4^ but have never been reported in combination with DLGNT.

## CASE PRESENTATION

Here we report the case of a 10-year-old Italian male, with a regular birth history and a diagnosis of autism spectrum disorder as well as normal intelligence and language (Asperger syndrome). Familiarity for migraine was reported in the maternal line (mother, grandmother, uncle), sporadically associated with sensory aura in the mother.

**FIGURE 1:**
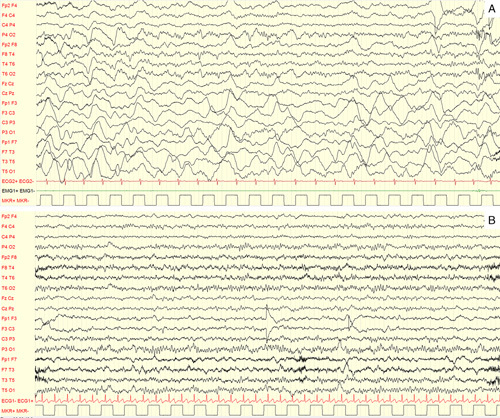
Electroencephalogram during the attack and after recovery. The first electroencephalogram, performed at the highest point of symptom severity (A), shows interhemispheric asymmetry with widely greater and slower activity in the left hemisphere. After 10 days the electroencephalogram has completely normalized, along with the disappearance of symptoms (B). Note: low frequency filter: 1 Hz; high frequency filter: 50 Hz; paper speed: 20 mm/s; sensitivity: 100 microV/cm.

**FIGURE 2:**
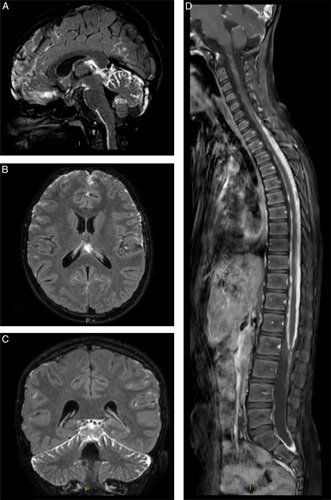
Brain and spinal MRI. Postcontrast T2-weighted 3D FLAIR fat-suppressed sequences, reconstructed in the sagittal (A), axial (B), and coronal (C) planes. MRI scan demonstrates diffuse leptomeningeal enhancement, most prominent within basal cisterna, posterior fossa, and along frontal and interhemispheric cerebral spaces. Sagittal T1-weighted postcontrast fat-suppressed image (D) shows thick leptomeningeal enhancement, predominantly along the dorsal thoracic spinal cord. MRI indicates magnetic resonance imaging.

He acutely presented a nocturnal awakening due to a frontal, pulsating, severe headache, associated with abdominal pain, rapidly followed by aphasia, repeated vomiting, and mild fever. He was then admitted to a pediatric emergency ward for further investigations. Nonenhanced brain computer tomography, magnetic resonance imaging (MRI), and ophthalmologic evaluation were unremarkable. Cerebrospinal fluid (CSF) analysis revealed increased proteins (312 mg/dL, reference 15 to 45) and polyclonal IgG (15.6 mg/dL, reference 0 to 4.5), impaired blood-CSF barrier permeability index (5.10, reference <4.9), mildly increased glucose (105 mg/dL, reference 50 to 80), and normal total cell counts. Microscopic, cultural, and virologic examinations were unremarkable. He progressively recovered in 7 days and then was discharged. In the following 6 months he presented monthly episodes of self-limiting migraine, occasionally associated with confusion, right-sided paresthesia, and speech impairment. Then, a second major episode occurred, associated with vomiting, fever, encephalopathy, central VII right cranial nerve paresis, aphasia, and right-sided hemiplegia, with reduced deep-tendon reflexes. A new CSF analysis resulted substantially similar to the previous one. Brain computer tomography, blood tests, investigations for autoimmune encephalitis, and *Mycobacterium tuberculosis* showed no alteration. The patient was then referred to our pediatric neurology center. An electroencephalogram (EEG) revealed marked asymmetry with left hemispheric slowing (Fig. 1). Since he met the clinical criteria for HM,^2^ genetic analyses for FHM were conducted, revealing no mutations in CACNA1A, ATP1A2, SCN1A, and PRRT2. In the same days, a new brain and spine MRI revealed diffuse leptomeningeal enhancement involving predominantly the basal cisterns and posterior fossa, as well along the spinal cord and cauda equina; no CSF flow abnormality was noted (Fig. 2).

Subsequently, a cauda equina biopsy with intraoperative neurophysiological monitoring was performed. Histologic and immunohistochemical examination revealed a DLGNT without BRAF/KIAA1549 rearrangement.

HM-like symptoms healed progressively in 2 weeks as long as EEG normalization.

A strict monitoring approach was first adopted because of the low grading of the tumor and the sporadic and self-limiting nature of the HM-like episodes. A prophylactic therapy with flunarizine 5 mg was initiated and only one self-limited HM-like attack occurred 10 days after discharge. However, the 2-month follow-up brain and spine MRI displayed an increased leptomeningeal contrast enhancement (especially in basal subarachnoid cisterns and cerebellar folia). The first cycle of treatment with vincristine and carboplatin was started according to standard therapy of pediatric oncology low-grade glioma (SIOP-LGG 2004).

In the following 3 months, the HM-like episodes lasting up to 3 to 4 days recurred with a frequency of about 1/month. At the follow-up MRI, tumor progression was detected, and hydrocephalus initially appeared, leading to ventricular-peritoneal shunt surgery. The postoperative period was characterized by the presence of a HM-like attack of considerable intensity. He also presented 2 close episodes in the following month. The therapy with flunarizine was replaced with topiramate+high-dose magnesium. Since then (4 mo) a discreet control of the headache has been maintained and no HM episode recurred. He is still undergoing chemotherapy treatment.

## DISCUSSION

Our patient presented with paroxysmal episodes of hemiplegia, aphasia, and headache that began acutely and led to repeated hospitalization because of their intensity and frequency, until the discovery of the DLGNT.

The episodes, while satisfying the ICDH3 criteria of HM, presented an unusually long duration and were poorly responsive to common symptomatic pharmacological therapies (acetaminophen, ibuprofen). Although considering the sure matrilineal predisposition to migraine, there was no familiarity for migraine aura, nor HM. Moreover, genetic investigations resulted unremarkable. This led us to continue diagnostic investigations.

Nevertheless, the diagnosis of DLGNT not associated with hydrocephalus at the beginning faced us with the question of whether to associate it with a symptom never reported before.

Migraine aura is known to be the result of a reversible cerebral cortical dysfunction secondary to cortical spreading depression (CSD): a short neuronal excitation starts a depolarization wave that moves through the cortex followed by prolonged inhibition of neuronal activity. This activates multiple loops involving the trigeminal system, the neurovascular system, and neuroinflammatory mechanisms that feed each other.^5^,^6^ In HM these mechanisms seem to be emphasized and perpetuated for a genetically determined susceptibility. Animal models of monogenic forms of FHM have highlighted how the known mutations increase neuronal excitability and reduce the threshold for CSD.^7^

In our patient, EEG slow waves in the hemisphere contralateral to hemiplegia indirectly confirm CSD and point toward a mechanism related to HM.

In the literature “HM-like attacks,” defined as episodes similar (for characteristics and complete interictal remission of symptoms) to HM^2^ have been previously described in the case of leptomeningeal involvement hypothesizing a pathogenesis overlapping with “idiopathic” HM. In Sturge Weber syndrome and cerebral autosomal dominant arteriopathy with subcortical infarcts and leukoencephalopathy vasomotor disturbances are responsible for the acute reduction of cerebral blood flow (oligoemia) which triggers CSD.^8^,^9^ Moreover, in Sturge Weber syndrome an impaired cortical circulation and a subsequential electrical cortical instability increased the sensitivity to CSD.^10^ In stroke-like migraine attacks after radiation therapy syndrome, HM-like episodes are a consequence of neuronal and endothelial damage/dysfunction and subsequent vasospasm which triggers the same cascading mechanisms. Some authors hypothesize that susceptibility to stroke-like migraine attacks after radiation therapy could involve genes associated with HM and this could explain why for the same radiation exposure only some patients develop the syndrome.^11^

In headache and neurological deficits with CSF lymphocytosis syndrome, symptomatology appears to be the result of an immune-mediated response triggered by a viral infection, secondarily inducing aseptic vasculitis and transient neurological symptoms by CSD mechanisms.^2^,^12^

To our knowledge, a single case of HM-like episodes related to a central nervous system tumor is reported in the literature as the first symptom of a diffuse leptomeningeal melanocytosis.^13^

Like leptomeningeal melanocytosis, DLGNT determines a widespread leptomeningeal infiltration and an alteration of the liquor dynamics secondary to subarachnoid obstruction. In both that case and our case, alteration of the blood-brain barrier is evidenced by elevation of the blood-CSF barrier permeability index.

There are no specific investigations in the literature studying the microenvironmental changes caused by DLGNT given its very recent identification as a nosological entity. However, glioneuronal tumors are known to alter both structurally and functionally the surrounding brain tissue. Edema and vascular insufficiency can directly interfere with physiological brain activity. The release of metabolically active molecules (such as those connected to interleukine-1β) leads to a proinflammatory state; a prevalence of glutamatergic activity results in hyperexcitability.^14^ Interestingly, many of these mediators and mechanisms are assumed to be triggers of migraine aura pathways noted above.

On these bases, we hypothesize that in our migraine-prone patient, the DLGNT, acting on the one hand through vasomotor mechanisms and on the other hand through the direct production of proinflammatory cytokines, lowers the CSD threshold resulting in HM-like attacks. The episodes had an increscent course, simultaneously with tumor progression, as well as triggered by neurosurgical intervention, itself irritating and proinflammatory.

**FIGURE 3:**
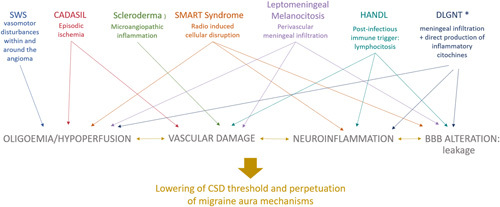
Reported causes of symptomatic hemiplegic migraine with leptomeningeal involvement and assumed mechanisms. The image summarizes the overlapping mechanisms suggested in the literature for the various conditions. All of these ultimately activate cortical spreading depression and trigger the hemiplegic migraine-like attack.^8^–^13^,^15^ *Mechanism hypothesized by the authors (of this article). BBB indicates blood brain barrier; CADASIL, cerebral autosomal dominant arteriopathy with subcortical infarcts and leukoencephalopathy; CSD, cortical spreading depression; DLGNT, diffuse leptomeningeal glioneuronal tumor; HANDL, headache and neurological deficits with cerebrospinal fluid lymphocytosis; SMART, stroke-like migraine attacks after radiation therapy; SWS, Sturge Weber syndrome.

Broadening the discussion to HM-like episodes in general, it could thus be suggested that, as in idiopathic forms specific genetic alterations (mostly ion channel) facilitates the development of prolonged focal signs,^1^,^5^,^6^ so in these “symptomatic” forms the underlying disease could act as an activator of the same pathways through the direct induction of vascular and/or inflammatory changes (Fig. 3).

## References

[1] Di Stefano V Rispoli MG Pellegrino N . Diagnostic and therapeutic aspects of hemiplegic migraine. J Neurol Neurosurg Psychiatry. 2020;91:764–771. 32430436 10.1136/jnnp-2020-322850 PMC7361005

[2] Olesen J . Headache Classification Committee of the International Headache Society (IHS) The International Classification of Headache Disorders, 3rd edition. Vol. 38, Cephalalgia. 2018:1–211. 10.1177/0333102417738202 29368949

[3] Chen W Kong Z Fu J . Diffuse leptomeningeal glioneuronal tumour (DLGNT) with hydrocephalus as an initial symptom: a case-based update. Child’s Nerv Syst. 2020;36:459–468. 31897629 10.1007/s00381-019-04481-w

[4] Vetvik KR Dahl M Russell MB . Symptomatic sporadic hemiplegic migraine. Cephalalgia. 2005;25:1093–1095. 16232163 10.1111/j.1468-2982.2005.00975.x

[5] Russell MB Ducros A . Sporadic and familial hemiplegic migraine: pathophysiological mechanisms, clinical characteristics, diagnosis, and management. Lancet Neurol. 2011;10:457–470. 21458376 10.1016/S1474-4422(11)70048-5

[6] Close LN Eftekhari S Wang M . Cortical spreading depression as a site of origin for migraine: role of CGRP. Cephalalgia. 2019;39:428–434. 29695168 10.1177/0333102418774299 PMC7007998

[7] Dehghani A Karatas H . Mouse models of familial hemiplegic migraine for studying migraine pathophysiology. Curr Neuropharmacol. 2019;17:961–973. 31092180 10.2174/1570159X17666190513085013 PMC7052833

[8] Planche V Chassin O Leduc L . Sturge-Weber syndrome with late onset hemiplegic migraine-like attacks and progressive unilateral cerebral atrophy. Cephalalgia. 2014;34:73–77. 24045571 10.1177/0333102413505237

[9] Rossi G Shambhu S . Hemiplegic migraine as the initial presentation of biopsy positive cerebral autosomal dominant arteriopathy with subcortical infarcts and leukoencephalopathy. Cureus. 2018;10:e2631. 30027023 10.7759/cureus.2631 PMC6044483

[10] Freilinger T Peters N Rémi J . A case of Sturge-Weber syndrome with symptomatic hemiplegic migraine: clinical and multimodality imaging data during a prolonged attack. J Neurol Sci. 2009;287:271–274. 19733861 10.1016/j.jns.2009.08.050

[11] Armstrong AE Gillan E Dimario FJ . SMART syndrome (stroke-like migraine attacks after radiation therapy) in adult and pediatric patients. J Child Neurol. 2014;29:336–341. 23364656 10.1177/0883073812474843

[12] Stelten BML Venhovens J Van Der Velden LBJ . Syndrome of transient headache and neurological deficits with cerebrospinal fluid lymphocytosis (HaNDL): a case report with serial electroencephalography (EEG) recordings. Is there an association with human herpes virus type 7 (HHV-7) infection? Cephalalgia. 2016;36:1296–1301. 26682576 10.1177/0333102415618616

[13] Brunsvig KL Zenobi M Rilliet B . Primary leptomeningeal melanocytosis in a 10-year-old girl: a challenging diagnosis with a poor prognosis. J Child Neurol. 2011;26:1444–1448. 21670389 10.1177/0883073811409749

[14] Loiacono G Cirillo C Chiarelli F . Focal epilepsy associated with glioneuronal tumors. ISRN Neurol. 2011;2011:1–6. 10.5402/2011/867503 PMC3263547 22389832

[15] Martins R Quintas S Coelho J . Extensive linear scleroderma en coup de sabre with exertion-induced hemiplegic migraine. Mult Scler Relat Disord. 2020;37:2019–2021. 10.1016/j.msard.2019.101457 31670009

